# Small Chemical Chromatin Effectors Alter Secondary Metabolite Production in *Aspergillus clavatus*

**DOI:** 10.3390/toxins5101723

**Published:** 2013-10-07

**Authors:** Christoph Zutz, Agnieszka Gacek, Michael Sulyok, Martin Wagner, Joseph Strauss, Kathrin Rychli

**Affiliations:** 1 Institute for Milk Hygiene, University of Veterinary Medicine Vienna, Veterinaerplatz1, Vienna 1210, Austria; E-Mails: christoph.zutz@vetmeduni.ac.at (C.Z.); martin.wagner@vetmeduni.ac.at (M.W.); 2 Fungal Genetics and Genomics Unit, Department of Applied Genetics and Cell Biology, University of Natural Resources and Life Sciences, Konrad Lorenz-Straße 24/II, Tulln/Donau 3430, Austria; E-Mails: agnieszka.gacek@boku.ac.at (A.G.); joseph.strauss@boku.ac.at (J.S.); 3 Center for Analytical Chemistry, Department for Agrobiotechnology (IFA-Tulln), University of Natural Resources and Life Sciences, Konrad-Lorenz-Straße 20, Tulln/Donau 3430, Austria; E-Mail: michael.sulyok@boku.ac.at; 4 AIT-Austrian Institute of Technology GmbH, Health and Environment Department, University and Research Campus Tulln, Konrad Lorenz-Straße 24/II, Tulln/Donau 3430, Austria

**Keywords:** secondary metabolites, mycotoxins, *Aspergillus clavatus*, HDAC inhibitors, 5-azacytidin, chromatin, nitrogen

## Abstract

The filamentous fungus *Aspergillus clavatus* is known to produce a variety of secondary metabolites (SM) such as patulin, pseurotin A, and cytochalasin E. In fungi, the production of most SM is strongly influenced by environmental factors and nutrients. Furthermore, it has been shown that the regulation of SM gene clusters is largely based on modulation of a chromatin structure. Communication between fungi and bacteria also triggers chromatin-based induction of silent SM gene clusters. Consequently, chemical chromatin effectors known to inhibit histone deacetylases (HDACs) and DNA-methyltransferases (DNMTs) influence the SM profile of several fungi. In this study, we tested the effect of five different chemicals, which are known to affect chromatin structure, on SM production in *A. clavatus* using two growth media with a different organic nitrogen source. We found that production of patulin was completely inhibited and cytochalasin E levels strongly reduced, whereas growing *A. clavatus* in media containing soya-derived peptone led to substantially higher pseurotin A levels. The HDAC inhibitors valproic acid, trichostatin A and butyrate, as well as the DNMT inhibitor 5-azacytidine (AZA) and *N*-acetyl-d-glucosamine, which was used as a proxy for bacterial fungal co-cultivation, had profound influence on SM accumulation and transcription of the corresponding biosynthetic genes. However, the repressing effect of the soya-based nitrogen source on patulin production could not be bypassed by any of the small chemical chromatin effectors. Interestingly, AZA influenced some SM cluster genes and SM production although no *Aspergillus* species has yet been shown to carry detectable DNA methylation.

## 1. Introduction

*Aspergillus* (*A.*) *clavatus* is a filamentous fungus, which is mainly isolated from soil and dung. *A. clavatus* plays a role in the spoilage of inadequately stored food products, such as rice, corn, and fruit juices, and produces a variety of mycotoxins like patulin, cytochalasin E and K, territrem B and brevianamid F [1].

Mycotoxins are typical secondary metabolites (SMs) produced by fungi mainly upon nutrient limitation and environmental stresses [2]. Furthermore, bacterial competitors and light do play an important role in the regulation of SM production [3,4,5]. Mycotoxins protect the fungi not only against other organisms, but also contribute to their virulence [2]. Genes involved in mycotoxin biosynthesis are usually organized in clusters and the majority is silenced during active growth [6,7,8]. The composition of the SM gene clusters is highly diverse, but every cluster contains at least a polyketide synthases (*PKS*s) and/or non-ribosomal peptide synthetase (*NRPS*) gene. For example, in *A. clavatus*, the patulin gene cluster consists of 15 genes, including the *PKS* gene *patK* [9], whereas the cytochalasin E gene cluster is composed of eight genes including one, *PKS-NRPS* gene [10].

The silencing of SM gene clusters has been linked to epigenetic mechanisms, which are responsible for the formation of “facultative heterochromatin”. This chromatin status restricts access to the underlying genetic material and thus results in reversible gene repression. Post-translational modifications of chromatin components, mainly histone acetylation and methylation, serve as signals for the recruitment or discharge of silencing or activating factors [11,12]. Histone acetylation, which is mainly linked to the activation of transcription, is controlled by the opposing actions of histone acetyltransferases (HATs) and deacetylases (HDACs) [13,14].

Recently it has been shown that various chemicals with low molecular masses, named in this study small chemical chromatin effectors (SCCEs), inhibit the catalytic activity of HDACs, mainly of class I and II, which can lead to a cryptic SM expression profile [15,16,17,18].

For instance, it has been shown that the anti-epileptic drug valproic acid (VPA) inhibits mainly the activity of class I HDACs, and induces additionally the proteosomal degradation of class II HDACs [19,20]. Other SCCEs such as trichostatin A (TSA), an antifungal compound produced by *Streptomyces* species [21,22] and HC-toxin, produced by *Cochliobolus carbonum* during maize leave infection, inhibits class I and class II HDAC activity [23]. Already in 1978 it was demonstrated that butyrate, a short chain fatty acid produced by anaerobic bacterial fermentation, induces differentiation of erythroleukemic cells via inhibition of HDACs [24]. However, the binding site of butyrate and the molecular mechanism remain unknown [25]. Overall, inhibition of HDAC activity and subsequent hyperacetylation of chromatin components leads to an open chromatin structure, reversal of heterochromatic landscapes to euchromatic structures and transcriptional activity in facultative heterochromatic regions [26].

An additional signal for the formation of heterochromatin and the subsequent inactivation of gene transcription is the methylation of cytidine catalyzed by DNA-methyltransferases (DNMTs). Inhibitors of DNMTs, among them 5-azacytidine (AZA), have been shown to increase the level of hypomethylated DNA leading to the expression of formerly silenced genes in different cell lines [18,27,28].

Recent findings indicate that the communication between microorganisms induces chromatin acetylation and the expression of silent gene clusters [29]. For example, the intimate physical interaction of *A. nidulans* with the soil-dwelling bacterium *Streptomyces rapamycinicus* increases histone acetylation and subsequently induces the production of several SM [5]. There is some evidence that *N*-acetyl-d-glucosamine (GlcNAc), which plays a key role in the structure of the extracellular surfaces of cells, can function as a proxy for bacterial and fungal co-cultivation [30]. Recently it was demonstrated that GlcNAc induced not only gene expression in *Candida albicans* [31], but also SM production in *Streptomyces* spp. [32].

In this study we analyzed the production of SM in *A. clavatus* in response to five different SCCEs. During our preliminary studies we noted that different organic sources of nitrogen used in a complex medium has a strong influence on the profile of SMs produced by *A. clavatus*. We therefore used two complex media with a different organic source of nitrogen and investigated the effect of VPA, TSA, butyrate, AZA, and GlcNAc on the production of patulin, cytochalasin E and pseurotin A, and the expression of the related *PKS*/*NRP*S genes, *patK* for the patulin, *ccsA* for the cytochalasin E and *psoA* for the pseurotin A gene cluster. We show that SCCEs alter the SM profile profoundly, but are not able to bypass the repressing effect of the soya-derived peptone in the growth broth.

## 2. Results

### 2.1. SM Production in *A. clavatus*

In this study, we used two different growth media with compounds commonly used for the cultivation of *Aspergillus* spp., e.g., organic nitrogen sources. Although the amount of peptone was identical in both media (2 g/L), we noted during the setup of the experiments that the source of peptone strongly influences the SM profile. We thus investigated the SM production of *A. clavatus* grown for 72 h in more detail in these two distinct media (termed FM1 and FM2). FM1 contains tryptic-digested casein peptone, which results in 0.27 g/L total nitrogen, whereas FM2 contains papaine-digested soya peptone resulting in a slightly smaller total nitrogen content of 0.20 g/L. This amount of supplied nitrogen corresponds approximately to a supplied amount of 5 mM glutamine, which is usually termed a “nitrogen sufficient” condition for growth and metabolism of *Aspergillus* spp. [33].

**Figure 1:**
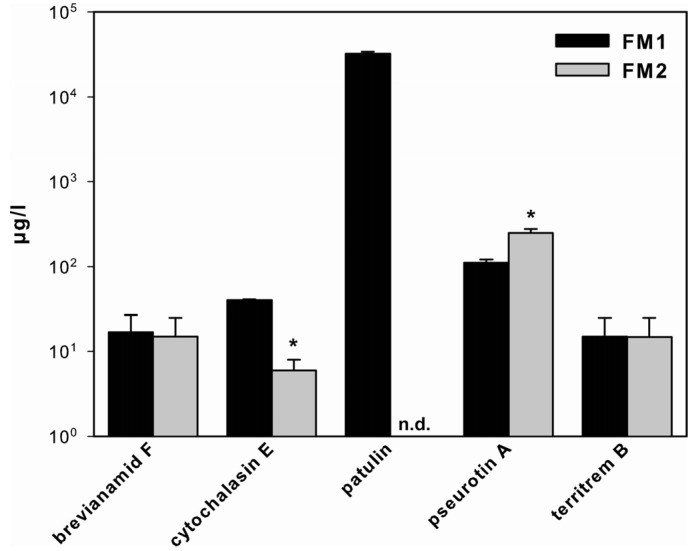
SM production in *A. clavatus.* Production of brevianamid F, cytochalasin E, patulin, pseurotin A and territrem B of *A. clavatus* grown for 72 h in FM1 (black bars) and FM2 (grey bars). Values are mean values ± standard deviation of three independent biological replicates, * indicates statistical significant difference between SM production in FM1 and FM2 (*p* < 0.05), n.d.: not detectable.

Unexpectedly, the difference in nitrogen source had a drastic effect on SM production (Figure 1). We detected five SMs: brevianamid F, cytochalasin E, patulin, pseurotin A and territrem B using a target based multi-mycotoxin method, which includes 186 fungal metabolites (Figure 1, note logarithmic scale of *y*-axis).

No differences were detected in the amount of brevianamid F and territrem B, but the production of cytochalasin E (6.7-fold lower in FM2) and pseurotin A (2-fold higher in FM2) strongly responded to the different nitrogen sources. However, the most striking effect was observed for patulin: using FM1 as growth media, 32 mg of this mycotoxin was produced, whereas when using FM2 patulin, it could not be detected at all (limit of detection: 5 µg/L).

### 2.2. Effect of SCCEs on SM Production in FM1

We next investigated the effect of SSCEs as single applications on SM production in *A. clavatus* grown for 72 h in FM1. In addition we applied the different SSCEs in combination with GlcNAc to investigate additive and synergistic effects on SM production.

**Figure 2:**
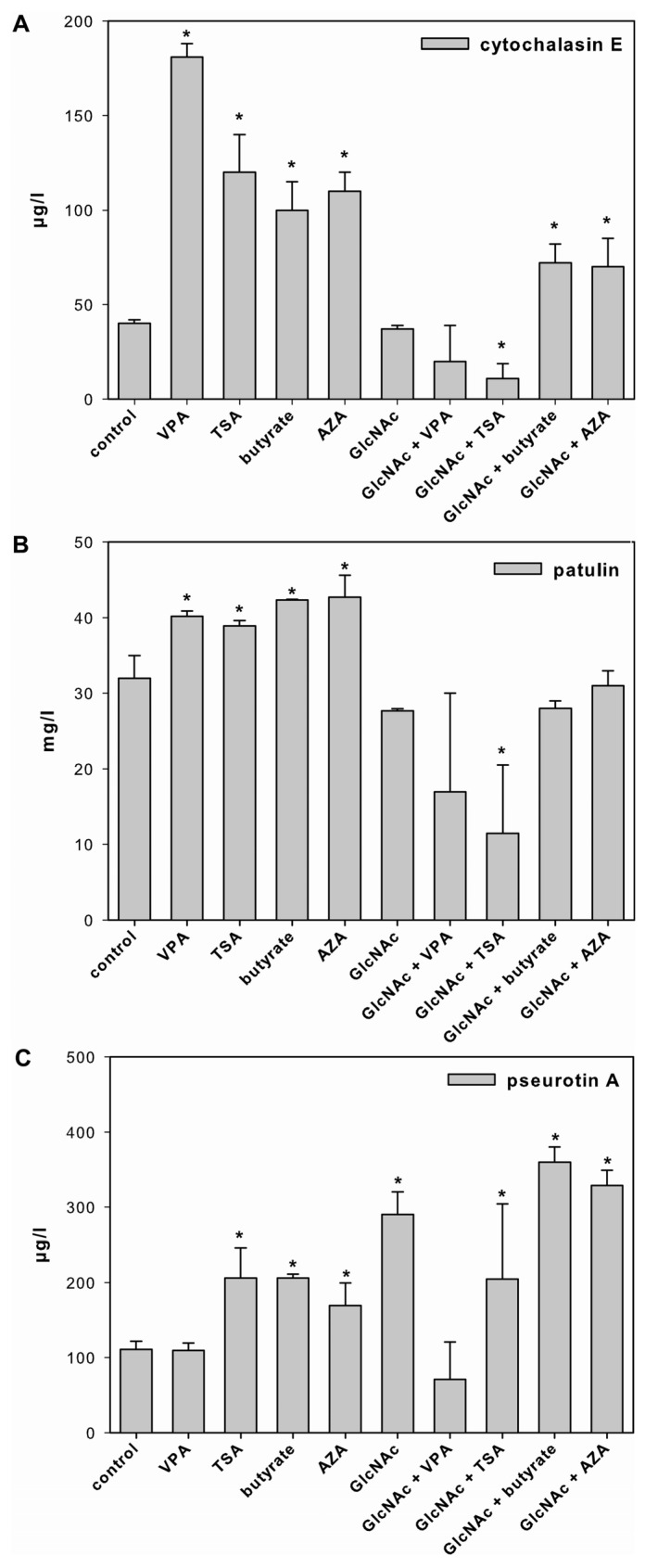
Effect of SCCEs on SM production. Cytochalasin E (panel **A**), patulin (panel **B**) and pseurotin A (panel **C**) production in *A. clavatus* grown for 72 h in FM1 in the absence (control) and presence of VPA, TSA, butyrate, AZA, and GlcNAc alone or in combination with VPA, TSA, butyrate and AZA. Values are mean values ± standard deviation of three independent biological replicates, * indicates statistical significant difference compared to the control (*p* < 0.05).

VPA, TSA, butyrate and AZA significantly increased the production of cytochalasin E and patulin, compared to the control (Figure 2A,B). Furthermore, the amount of pseurotin A was significantly higher if *A. clavatus* was grown in the presence of TSA, butyrate, AZA and GlcNAc. In addition, the combinations of GlcNAc with butyrate and AZA showed an additive effect on the pseurotin A production (Figure 2C and Figure S1).

### 2.3. Effect of SCCEs on SM Production and PKS Gene Expression in FM2

Because FM2 suppressed the production of patulin and cytochalasin E, but stimulated pseurotin production, we investigated the effect of the different SCCEs in FM2 at two different time points (48 h and 72 h). In parallel, we analyzed the effect of these SCCEs on the expression of the related *PKS* genes, *cssA* for cytochalasin E, *patK* for patulin and *psoA* for pseurotin A.

**Figure 3:**
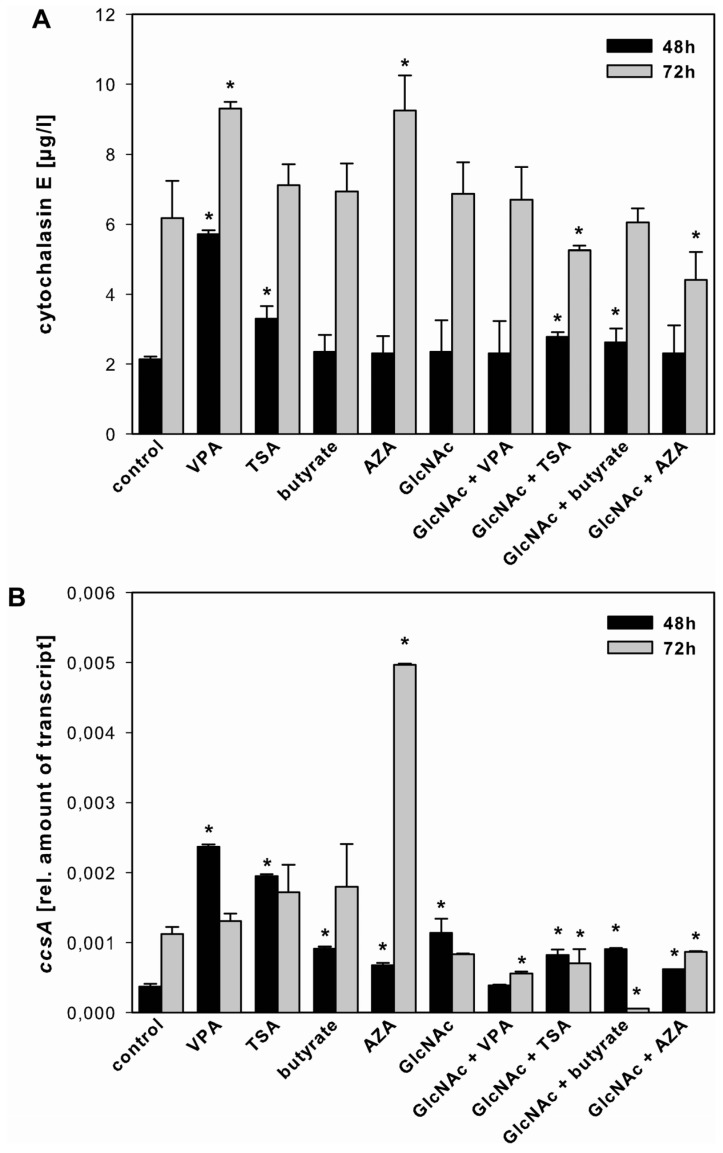
Effect of SCCEs on cytochalasin E production and *ccsA* expression. Cytochalasin E production (panel **A**) and *ccsA* expression (panel **B**) in *A. clavatus* grown for 48 h (black bars) and 72 h (grey bars) in FM2 in the absence (control) and presence of VPA, TSA, butyrate, AZA, and GlcNAc alone or in combination with VPA, TSA, butyrate and AZA. Values are mean values ± standard deviation of three independent biological replicates, * indicates statistical significant difference compared to the control (*p* < 0.05).

#### 2.3.1. Cytochalasin E

Using FM2, VPA significantly increased the production of cytochalasin E after 48 and 72 h, TSA only after 48 h, whereas the presence of AZA resulted in a higher amount of cytochalasin E only after 72 h (Figure 3A). Consistent with metabolite accumulation, the expression of the biosynthetic *ccsA* gene was highest in the presence of VPA and TSA after 48 h, and in the presence of AZA after 72 h (Figure 3B and Figure S2). In addition, the combination of GlcNAc with TSA or butyrate slightly increased the production of cytochalasin E and the expression of *ccsA* after 48 h.

#### 2.3.2. Patulin

**Figure 4:**
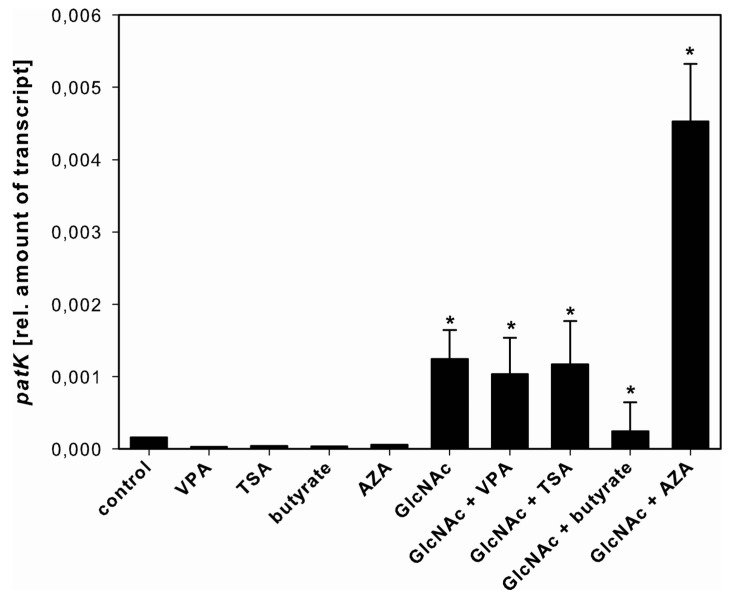
Effect of SCCEs on *patK* expression. *PatK* expression in *A. clavatus* grown for 48 h in FM2 in the absence (control) and presence of VPA, TSA, butyrate, AZA, and GlcNAc alone or in combination with VPA, TSA, butyrate and AZA. Patulin production was not detectable after 48 and 72 h, and no *patK* expression was detectable after 72 h. Values are mean values ± standard deviation of three independent biological replicates, * indicates statistical significant difference compared to the control (*p* < 0.05).

On FM2, even the presence of SCCEs cannot bypass the repressive effect of this medium as under all conditions the amount of patulin was below the limit of detection (data not shown). Interestingly, the expression of *patK* was already detectable after 48 h in the control cultures, though no metabolites were produced. In addition, GlcNAc alone or in combination with all other SSCEs significantly induced the expression rate of *patK*; however, no patulin was detectable (Figure 4, Table S2). Moreover, no *patK* expression was detectable after 72 h.

#### 2.3.3. Pseurotin A

**Figure 5:**
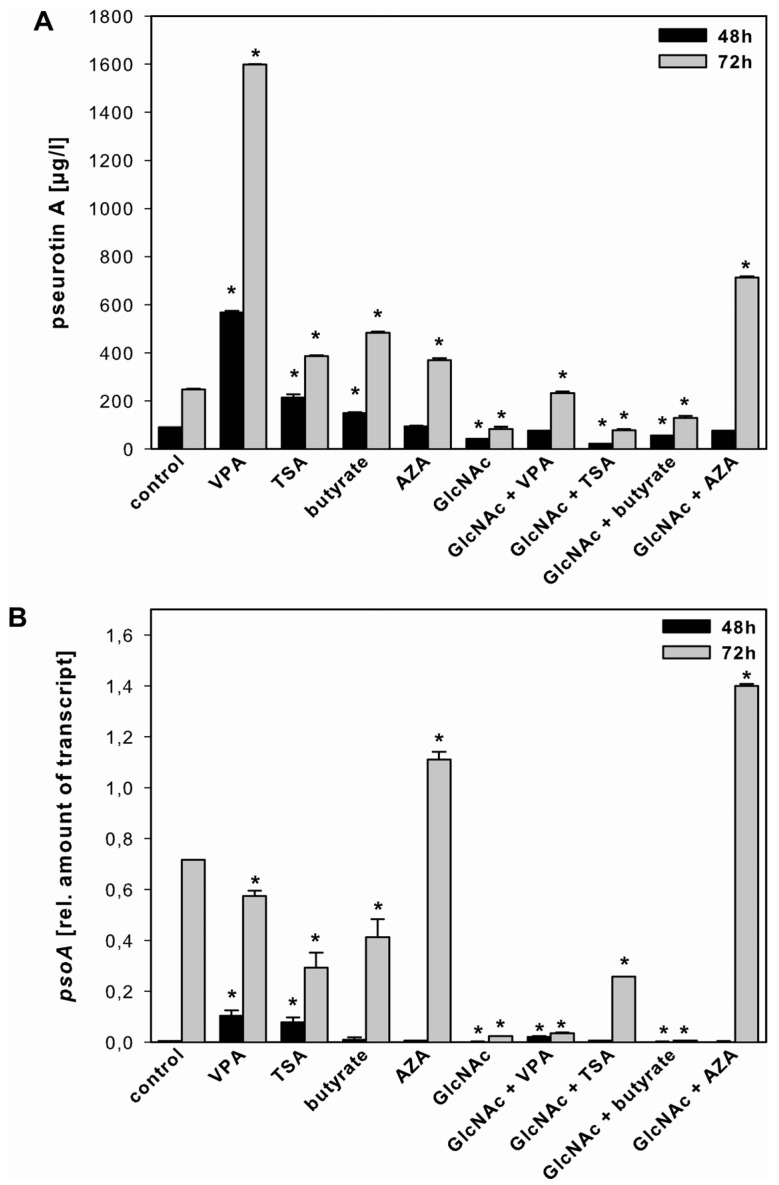
Effect of SCCEs on pseurotin A production and *psoA* expression. Pseurotin A production (panel **A**) and *psoA* expression (panel **B**) in *A. clavatus* grown for 48 h (black bars) and 72 h (grey bars) in FM2 in the absence (control) and presence of VPA, TSA, butyrate, AZA, and GlcNAc alone or in combination with VPA, TSA, butyrate and AZA. Values are mean values ± standard deviation of three independent biological replicates, * indicates statistical significant difference compared to the control (*p* < 0.05).

The amount of pseurotin A increased up to 6.5-fold in the presence of VPA, TSA and butyrate both after 48 and 72 h, whereas AZA increased pseurotin A production, alone or in combination with GlcNAc only after 72 h (Figure 5A). In addition, we detected lower amounts of pseurotin A if *A. clavatus* was grown for 48 and 72 h in the presence of GlcNAc alone or in combination with TSA and butyrate.

The overall expression of the related *PKS* gene *psoA* was low after 48 h, although it was upregulated in the presence of VPA and TSA. After 72 h, only AZA, either alone or in combination with GlcNAc, increased the expression of *psoA* (Figure 5B, Table S2).

## 3. Discussion

### 3.1. SM Production in *A. clavatus*

The filamentous fungus *A. clavatus* produces a variety of SM, some of them with strong toxic effects [1,34]. Employing a quantitative liquid chromatography/tandem mass spectrometry method, which detects simultaneously 186 microbial metabolites [35], we detected five compounds in the supernatant of *A. clavatus* grown on a semi-synthetic medium with yeast extract and peptone as organic nitrogen sources: brevianamid F, cytochalasin E, patulin, pseurotin A and territrem B. However, kojic acid, a primary metabolite known to be produced by *A. clavatus*, was not detected under these growth conditions probably due to inappropriate media components or inadequate pH, which should be around pH 3–4 for optimal kojic acid production [36].

The SMs kotanin, alpha-sarcin and glyantripine, which are also produced by *A. clavatus* [1], have not been included in the quantitative multi-mycotoxin detection method due to the lack of authentic standards.

### 3.2. Growth Media-Dependent Production of SM

It is known that the production of SM in fungi is influenced by temperature, light, pH and the availability of nutrients and oxygen [37,38,39,40,41,42,43,44]. On a broad regulatory level, these signals change the chromatin structure and, in addition, each biosynthetic pathway is regulated by specific activators and repressors [45].

In this study, we tested whether compounds with known chromatin-modifying effects alter the metabolite profile of *A. clavatus* and if the type of nitrogen influences the effect of the chemical chromatin effectors. All other parameters, such as light, pH, carbon source and trace elements, have been kept constant. FM1 contained tryptic-digested peptone from casein (final nitrogen concentration 0.27 g/L), whereas FM2 contained papainic-digested peptone from soya (final concentration 0.20 g/L). The chosen amount of organic nitrogen supported regular fungal growth and can therefore be regarded as a “nitrogen sufficient” condition in terms of nitrogen metabolism. Our data suggest that the type of nitrogen strongly affected SM production. The amount of cytochalasin E, a potent anti-angiogenic compound, was 7-fold lower in FM2. An older study by Vesley *et al*. [46] reported that only one third of all tested *A. clavatus* strains produced cytochalasin E at all. Additionally, the cytochalasin E production under different fermentation conditions has been extensively studied [47], but the effects of single abiotic factors like nitrogen have not yet been investigated.

In addition, we recognized that the production of brevianamid F, a precursor for fungal prenylated alkaloids like fumitremorgins [48] and territrem B, a tremorgenic toxic compound [49], was independent of the availability and source of nitrogen. In contrast, the amount of pseurotin A, a competitive inhibitor of chitin synthase and neuritogenic agent [50,51], is higher if FM2 was used as a growth media. The influence of nitrogen on pseurotin A production is yet to be investigated.

Furthermore, the production of patulin, which induces immunological, neurological and gastrointestinal disorders [1], was completely inhibited if *A. clavatus* was grown in the soya-based nitrogen medium FM2. The influence of the amount and the type of carbon and nitrogen source (organic or inorganic) on patulin production has been previously reported; Grootwassink, for instance, showed in *Penicillium* spp. that low-level nitrogen induced patulin production [52], and Stott [53] demonstrated in *P. patulum* that nitrate and glucose result in a high amount of patulin. He also determined that organic nitrogen sources like peptonized milk together with glucose lead to a decreased amount of patulin. The total lack of measurable patulin in FM2 indicates that a soya-based nitrogen source is not suitable for patulin production in *A. clavatus*.

Nitrogen regulation of SM production is a known phenomenon, but it is still poorly understood at the molecular level [54,55,56,57,58]. For example, *A. nidulans* produces the aflatoxin precursor sterigmatocystin only if nitrate serves as a nitrogen source, whereas *A. flavus* produces the highly similar aflatoxin only with ammonium as the nitrogen source [59].

A more detailed molecular picture can be drawn from nitrogen regulation of bikaverin and gibberellin biosynthesis in the plant pathogen *Fusarium fujikuroi*. In this fungus, the main nitrogen regulator AreA, which is best studied in *A. nidulans* [54], has been shown to be required for SM production [60]. Whether or not an AreA ortholog also regulates SM production in *A. clavatus* remains to be shown.

Recent evidence indicates that in *Aspergillus* spp. there are, besides AreA, many regulatory components involved in the transcription of mycotoxin biosynthesis genes, including regulators involved in chromatin formation, e.g., LaeA and pathway-specific activators [45,61]. Alongside gene cluster-specific transcription regulators, which are responsible for the regulation of mycotoxin biosynthesis, the complexity of the biosynthesis pathway could be an additional reason for the different mycotoxin profiles. In *A. clavatus*, the gene cluster of patulin consists of 15 genes, whereas only 8 genes are involved in biosynthesis of cytochalasin E [9]. The gene cluster of pseurotin A, thus far only characterized in *A. fumigates*, is composed of 6 genes [62,63].

### 3.3. Effect of SCCEs on SM Production

All five SCCEs showed a strong effect on the production of SM, but the effects were metabolite-specific and depended on the growth media and the incubation time.

If the fungus was grown for 72 h in FM1, the media with the slightly higher amount of total nitrogen from casein, all SCCEs except for GlcNAc, increased the amount of cytochalasin E and patulin. Pseurotin A production, however, was increased in the presence of all SCCEs except for VPA.

The effect of the SCCEs was different if FM2 was used as a growth media. After 72 h, cytochalasin E production increased—although to a lesser extent when exclusively in the presence of VPA and AZA—while patulin production was not detectable, and the amount of pseurotin A was higher in the presence of all SCCEs except for GlcNAc.

This can be interpreted in two ways: (i) it is known from previous studies that different clusters respond differently to changes in the chromatin landscape [45]; and (ii) alternatively, or additionally, the function of cluster-specific transcriptional regulators might be affected differently by the SCCEs. Another level of complexity is brought in by the fact that chromatin regulation, e.g., mediated by the LaeA protein which counteracts heterochromatin formation in *A. nidulans* and *F. graminearum*, directly regulates the expression of the pathway-specific transcriptional activators, such as aflR [64]. This regulation is essential also for the production of SMs like penicillin and sterigmatocystin in *A. nidulans* and gliotoxin in *A. fumigatus* [64,65,66,67]. In addition, the homolog of LaeA regulates in *P. chrysogenum* the biosynthesis of penicillin, but not roquefortin C biosynthesis [68].

Our results show that inhibitors of class I and class II HDACs, mainly VPA and TSA, induced already cytochalasin E and pseurotin A production after 48 h, in the early phase of secondary metabolism. Similarly, the expression of the related PKS genes *ccsA* and *psoA* increased after 48 h, but subsequently decreased after 72 h. We speculate that chromatin acetylation of SM gene clusters might occur to a certain extent already during primary metabolism, which has already been shown for the sterigmatocystin cluster of *A. nidulans* [67]; otherwise, HDAC inhibitors would not have such a strong influence after 48 h. However, more experiments including chromatin immunoprecipitation of acetylated histones at the tested metabolite gene clusters are needed to evaluate the time-dependent effect of HDAC inhibitors on mycotoxin production.

The stability of VPA, TSA and butyrate in a fungal culture could also play a role in the regulation of SM production. Butyrate, which has only a modest effect, is a naturally occurring short-chain fatty acid used as an anti-cancer drug. In patients, the half-life of butyrate is short [69]; therefore the stability in a fungal culture could also be limited.

In contrast to the HDAC inhibitors, the effect of AZA was detectable only after 72 h of growth. AZA incorporates into the DNA and inhibits DNMT, leading to a reduced methylation level of genomic DNA. Although different studies showed that AZA changed the SM profile in various *Aspergillus* spp*.* [15,70,71], there are indications that the DNA methylation level in *Aspergillus* spp. is negligible [72,73]. In addition, Lee *et al*. demonstrated in *A. nidulans* that the solely DNMT-homolog DmtA is involved in sexual development. There is growing evidence that AZA is also incorporated into RNA, leading to destruction of nucleic acid and protein metabolism [74]. In addition, AZA could also inhibit the activity of RNA methyltransferases. RNA methylation has been detected in both prokaryotic and eukaryotic noncoding RNAs, although the function of RNA methylation still remains to be elucidated [75]. A recent study illustrated by investigating the transcriptome of *A. flavus* that AZA increased only one SM biosynthesis gene cluster [76]. However, the expression of various genes involved in development were changed [76], such as the AZA that decreased the expression of *veA*, which is one major regulator of light response, development, and SM [77]. In various *Aspergillus* spp., it was shown that VeA, which also interacts with LaeA, is essential for SM production [78]. However, more studies are essential to elucidate the mode of action of AZA in organisms lacking DNMTs. GlcNAc, which functions as a proxy for bacterial and fungal co-cultivation and potentially increases histone acetylation, affected only the production of pseurotin A. Notably, the amount of pseurotin A was increased in FM1, but decreased in FM2. Pseurotin A is known to inhibit the biosynthesis of chitin [51]. Chitin, a polymer of GlcNAc, is not only present in lower invertebrates and insects, but also in microbes including fungi, protists and algae [79]. Our data show that in *A. clavatus* the chitin component GlcNAc is involved in the regulation of pseurotin A production. The combination of AZA and GlcNAc resulted in FM1 in an additive effect on pseurotin A production, whereas in FM2, AZA reversed the inhibiting effect of GlcNAc on pseurotin A production. However, the reason why the different source of nitrogen has such a strong influence on the effect of GlcNAc alone and in combination with AZA is as yet still unclear.

Additionally, we also show that although GlcNAc induces the expression of the *PKS* genes *patK* and *cssA* after 48 h, no increased mycotoxin production was detectable. If *A. clavatus* was grown in FM2, no patulin was detectable, although the expression of *patK* expression was induced in the presence of GlcNAc alone or in combination with the other SCCEs. The patulin biosynthesis consists of ten steps. The *NRPS*-*PKS* hybrid *patK* is involved in the first step and produces the first intermediate compound [80]. Our results indicate that the induction of the *NRPS-PKS* gene is not sufficient for the biosynthesis of this mycotoxin under these conditions. However, more detailed expression studies are essential to explicate the regulation of patulin biosynthesis.

## 4. Experimental Section

### 4.1. Fungal Strain and Chemicals

The *A. clavatus* strain L19, which was used in this study, was obtained from the fungal strain collection of the AIT [81]. The conidial spores of the fungus were stored in glycerol buffer (PBS pH 7, 20% glycerol, Sigma Aldrich, St. Lois, MO, USA) at −80 °C. For inoculum preparation, spores were inoculated on malt extract agar (MEA, Oxoid, Cambridge, UK) and grown for 10 days at room temperature.

SCCE stock solutions, all obtained from Sigma-Aldrich, were prepared as followed: valproic acid (VPA, 2-propylpentanoic acid), 60 µM was dissolved in 96% ethanol. Trichostatin A (TSA, 7-4-(dimethylamino)phenyl-*N*-hydroxy-4,6-dimethyl-7-oxohepta-2,4-dienamide), 0.5 µM), sodium butyrate (butyrate, 9 µM), 5'-azacytidine (AZA, 4-amino-1-β-d-ribofuranosyl-1,3,5-triazin-2(1*H*)-1, 2 µM) and *N*-acetyl-d-glucosamine (GlcNAc, 0.5 µM) were dissolved in PBS (pH 7). Stock solutions were stored at −20 °C for a maximum of 6 months.

### 4.2. Culture Conditions

Ten-day-old conidial spores were harvested from the stock plates by drenching the plate with 5 mL of PBS containing 0.1% Tween and scraping off the mycelium with a sterile cotton stick without damaging the agar. The spore solution was agitated for 1 min; spores were counted and inoculated in 20 mL fungal minimal media (FM) at a concentration of 10^6^ spores/mL. Two different FM were used, containing either tryptic-digested peptone from casein with a total nitrogen content of 0.25 g/L (FM1) or papainic-digested peptone from soya containing 0.18 g/L total nitrogen (FM2; Table 1). The pH of both media was set to 5.9 and reached under all conditions after the cultivation period the pH of 4.7.

Fungi were grown in the absence and presence of VPA, TSA, butyrate, AZA and GlcNAc either alone or in combination with GlcNAc (all final concentration of 5 µM) for 48 and 72 h at room temperature in the dark with orbital shaking (150 rpm). The fungal cultures were filtrated through a sterile cellulose filter. The supernatant was stored at −20 °C until mass spectrometric measurement. The mycelium was dried between two sheets of whatman filter paper (GE Healthcare, Pollards Wood, UK), frozen in liquid nitrogen and stored at −80 °C until RNA isolation. Each experiment was carried out in three independent replications.

**Table 1:** Composition of the fungal minimal media FM1 and FM2.

Components	FM1	FM2
Glucose (g/L)	10	10
KH_2_PO_4_ (g/L)	0.5	0.5
Inositol (g/L)	0.05	0.05
CaCl_2_ (g/L)	0.075	0.075
FeCl_3_ (g/L)	0.01	0.01
MgSO_4_ (g/L)	0.15	0.15
MnSO_4_ (g/L)	0.01	0.01
Yeast extract (g/L)	0.2	0.2
*nitrogen* (g/L)	*0.016*	*0.016*
Tryptic-digested peptone from casein (g/L)	2	
*nitrogen* (g/L)	*0.25*	
Papain-digested peptone from soya (g/L)		2
*nitrogen* (g/L)		*0.18*
*total nitrogen* (g/L)	*0.266*	*0.196*

### 4.3. Analysis of SM Production

We used a recently developed targeted multi-mycotoxin detection method based on liquid chromatography/electrospray ionization-tandem mass spectrometry (HPLC/ESI-MS/MS) for the detection of mycotoxins in the supernatant of *A. clavatus* [35]. SM analysis was performed according Vishwanath *et al*. [35]. Shortly after, samples were extracted with acetonitril/water/acetic acid, 79:20:1 (*v*/*v*/*v*) and chromatographic separation was performed on a Gemini^®^ C_18_ column with 150 × 4.6 mm in diameter, a particle size of 5 µm and a 4 × 3 mm in diameter security guard cartridge (Phenomenex, Torrance, CA, USA). Mycotoxins were detected with a QTrap 4000 LC-MS/MS System (Applied Biosystems, Framingham, MA, USA; 1100 Series HPLC systems Agilent, Waldbronn, Germany) and data were analyzed using Analyst^®^ software.

### 4.4. Transcriptional Analysis

For RNA isolation, the frozen mycelium was ground up to a fine powder. The RNA extraction was performed using TRIzol^®^ (Invitrogen, Carlsbach, CA, USA) following the established protocol for tissues. RNA concentration and purity was measured on a Nanodrop (Thermo Scientific, Waltham, MA, USA). RNA integrity was analyzed using a 0.8% agarose gel.

Reverse transcription quantitative real-time PCR (RT-qPCR) was used to determine the relative expression of the PKS genes of the biosynthesis gene clusters of cytochalasin E, patulin and pseurotin A (*ccsA* for cytochalasin E, *patK* for patulin and *psoA* for pseurotin A). Reverse transcription of RNA was performed using the iScript™ cDNA Synthesis Kit according to the manufacturer’s protocol (Biorad, Munich, Germany). Quickly, 1 µg of RNA was reverse-transcribed using a highly efficient RNase H+ MMLV reverse transcriptase and a mix of oligo dT and random hexamer primers.

We designed primers targeting the PKS genes according to the genome database of *A. clavatus* NRRL1 using the Primer 3 software [82]. *CcsA* transcription was assessed using the primers *ccsA_f*: CTCACGGAACATTGTTGTGG and *ccsA_r*: CCAAACGCCATAGGAACACT (product size: 320 bp), *patK* using patK_f: GATCCTCTCACCGTCCGATA, patK_r: GCAAGATACCCTTGCGGATA (product size: 219 bp), and *psoA* using psoA_f: GTGCACTGGACGAGGGTAAT, psoA_r: GCGGTACTCAATGCCGTTAT (product size: 168 bp). The primers were tested for target specificity and interlooping hairpin structure formation using Blast software.

For the RT-qPCR reaction, we diluted cDNA 1 to 100 with DEPC-ddH_2_O and used iQtm SYBR^®^ Green Supermix (Biorad, Munich, Germany) for amplification and detection. The following RT-qPCR conditions were used: initial denaturation at 95 °C for 5 min; 50 cycles of denaturation at 95 °C for 15 s, annealing at 63 °C for 30 s, elongation at 72 °C for 15 s; and final denaturation at 95 °C for 30 s (MyiQ cycler Hercules, Biorad, Munich, Germany). Input normalization of transcript levels was performed by comparison to transcript levels of the housekeeping gene actin (*acnA*_f: GTACCACCGGTATCGTGCTT; *acnA*_r: CTCAGCGGTAGTGGAGAAGG; product size: 176 bp). As internal controls, standard curves using serial dilutions of genomic DNA from *A. clavatus* and water as a negative control were included.

### 4.5. Statistical Analysis

Microsoft Excel^®^ 2007 was used for statistical analysis. To study the effect of the different treatments, values were compared statistically using *t*-test (independent variables). *p*-values < 0.05 were considered to be significant.

## 5. Conclusions

In this study, we demonstrate that the source of nitrogen has a tremendous effect on production of SM in *A. clavatus*. We assume that peptone derived either from casein or soya provide different compounds, which could be used as precursors for the SM production and therefore result in different SM profiles. Further experiments are needed to exclude the presence of repressing compounds in the different nitrogen sources.

In addition, all SCCEs, HDAC inhibitors, as well as the DNMT inhibitor AZA and the chitin component GlcNAc, influenced SM biosynthesis in a media-, time- and target-dependent way. Furthermore, our data suggest that different regulatory components are involved in the transcription of mycotoxin biosynthesis genes, including pathway-specific activators and epigenetic regulators.

## Supplementary Files

Supplementary File 1 Supplementary Information (PDF, 81 KB)
