# Supplementary material for: Small Chemical Chromatin Effectors Alter Secondary Metabolite Production in Aspergillus clavatus
Source: Toxins (Basel). 2013 Oct 7;5(10):1723–41. doi: 10.3390/toxins5101723 (PMC3813908; doi:10.3390/toxins5101723)
Supplement: Supplementary File 1 — Supplementary Information (PDF, 81 KB) [file toxins-05-01723-s001.pdf]

Supplementary Information

Figure S1. Effect of SCCEs on SM production in *A. clavatus* grown for 72 h in FM1. Values are x-fold of control.***** indicates significant increase and ° means significant decrease compared to the control.

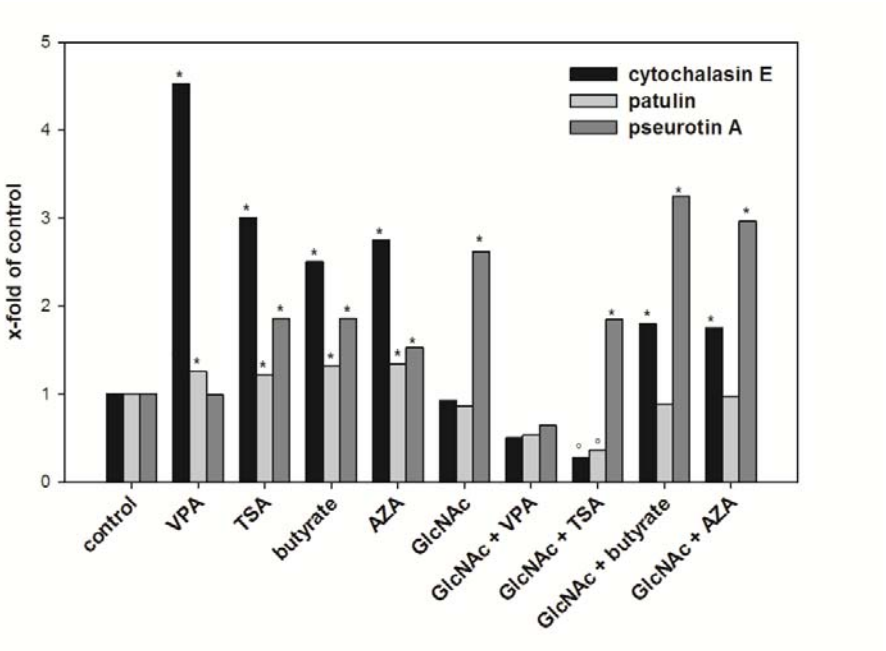

Figure S2. Effect of SCCEs on SM production and expression of the related PKS-gene in *A. clavatus* grown for 48 and 72h in FM2. Effect of SCCEs on cytochalasin E production and *ccsA* expression (panel **A**), on patulin production and *patK* expression (panel **B**) and on pseurotin A production and *psoA* expression (panel **C**). Values are x-fold of control. *****indicates significant increase and °means significant decrease compared to the control.

A

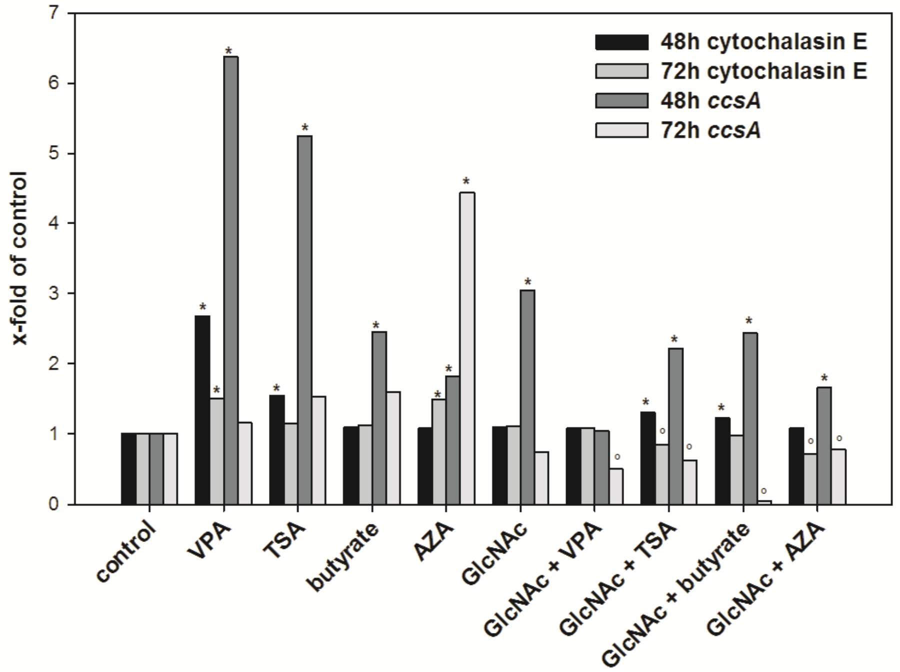

Figure S2. *Cont.*

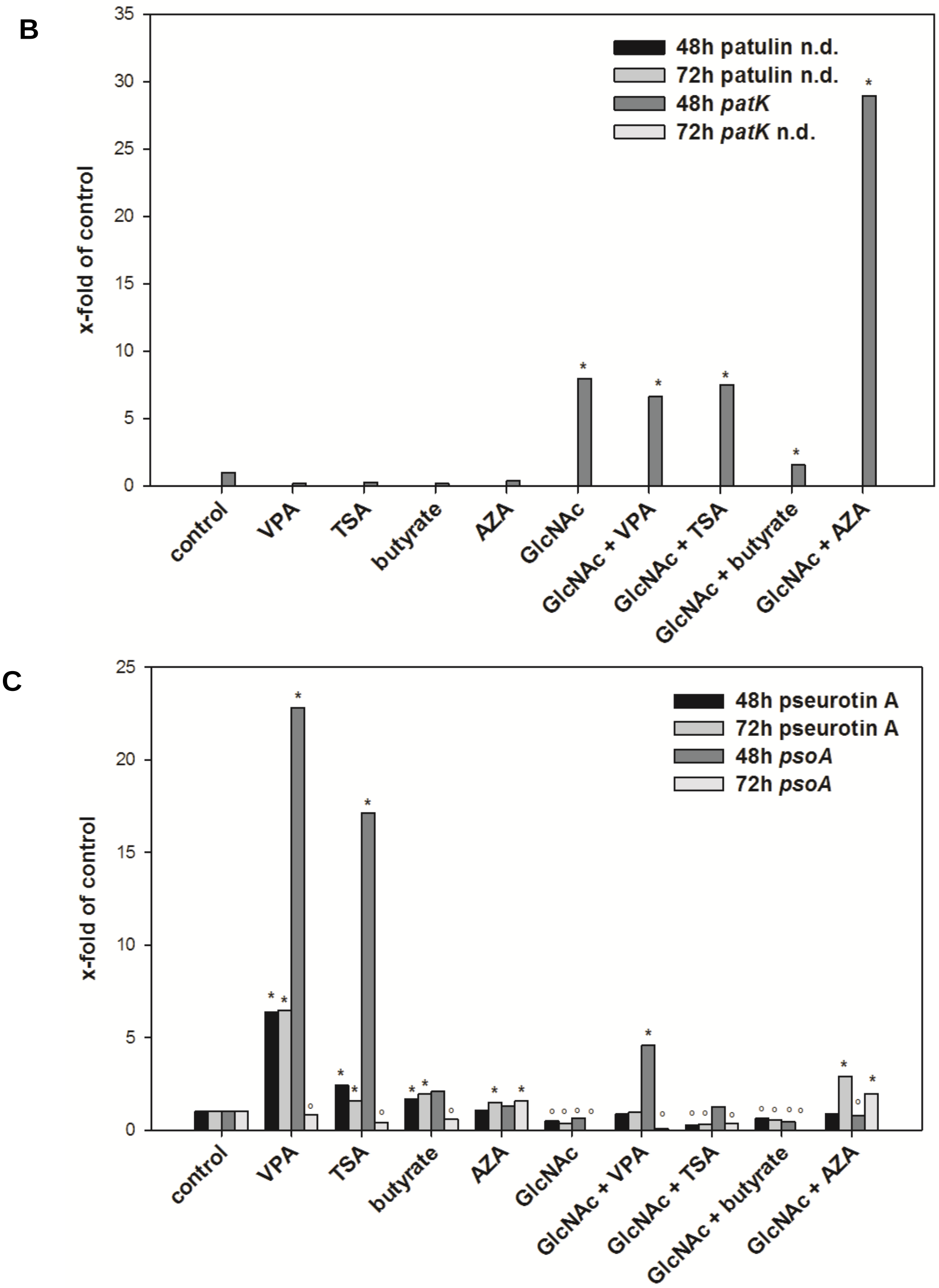

© 2013 by the authors; licensee MDPI, Basel, Switzerland. This article is an open access article distributed under the terms and conditions of the Creative Commons Attribution license (http://creativecommons.org/licenses/by/3.0/).
